# Acute median arcuate ligament syndrome after pancreaticoduodenectomy

**DOI:** 10.1186/s40792-018-0545-x

**Published:** 2018-11-26

**Authors:** Daisuke Imai, Takashi Maeda, Huanlin Wang, Takahiro Ohmine, Keitaro Edahiro, Makoto Edagawa, Tomoyoshi Takenaka, Shohei Yamaguchi, Kozo Konishi, Shinichi Tsutsui, Hiroyuki Matsuda

**Affiliations:** Department of Surgery, Hiroshima Red Cross Hospital and Atomic Bomb Survivors Hospital, 1-9-6, Sendamachi, Naka-ku, Hiroshima City, Hiroshima, 730-0052 Japan

## Abstract

**Background:**

Median arcuate ligament syndrome (MALS) can cause severe complications after pancreaticoduodenectomy (PD). Most of the reported cases of MALS have been diagnosed perioperatively and can be treated efficiently by interventional radiology or division of the median acute ligament (MAL) fibers.

**Case report:**

A 69-year-old woman underwent PD with resection of the SMV for pancreatic head cancer. Intraoperative exploration showed normal anatomy of the celiac trunk. Intraoperative digital palpation revealed normal pulsation of the common hepatic artery after resection of the gastroduodenal artery. On postoperative day (POD) 3, her liver function tests were abnormal, and bloody fluids were found in the drain. Abdominal CT showed necrosis of the pancreatic body and ischemia in the liver secondary to MALS which was not detected in the preoperative CT. Interventional radiology was tried first but failed. Division of the MAL fibers markedly increased the blood flow in the hepatic artery. Resection of the remnant pancreas and spleen was also performed simultaneously. Abdominal CT on POD 20 showed re-occlusion of the celiac artery. She experienced rupture of the gastrojejunostomy site, severe hepatic cytolysis, and choledochojejunostomy stricture thereafter.

**Conclusions:**

This is the third case of MALS that has developed acutely after PD. MALS can cause refractory complications even after MAL release.

## Introduction

The incidence of celiac axis stenosis caused by median arcuate ligament syndrome (MALS) is reported in around 7.3% of asymptomatic individuals [1]. MALS has been reported in from 2% to 7.6% of patients undergoing pancreaticoduodenectomy (PD) [2, 3].

The median arcuate ligament (MAL) is a normally occurring tendinous band spanning both diaphragmatic crura anterior to the aorta [2]. In MALS, this dense fibrous band causes extrinsic compression anteriorly on the celiac axis, leading to partial or complete celiac occlusion [2]. The pancreaticoduodenal arcades represent the largest collateral circle to allow retrograde flow through the gastroduodenal artery (GDA) if celiac occlusion occurs. Breakdown of this retrograde flow during PD in a patient with MALS causes serious complications due to ischemia of the liver, stomach, spleen, or pancreas [4].

Most of the reported cases of MALS have been diagnosed perioperatively, either by radiologic findings or by intraoperative digital palpation or Doppler sonography [2]. When MALS is diagnosed preoperatively, it can be treated by interventional radiology, MAL division, or bypass grafting [5, 6]. These strategies have been reported to be usually effective in restoring an adequate blood flow and preventing ischemic complications [7, 8].

Herein, we report a case of MALS that developed acutely after PD that was refractory to interventional radiology and MAL division, leading to serious ischemic complications.

## Case presentation

**Fig. 1:**
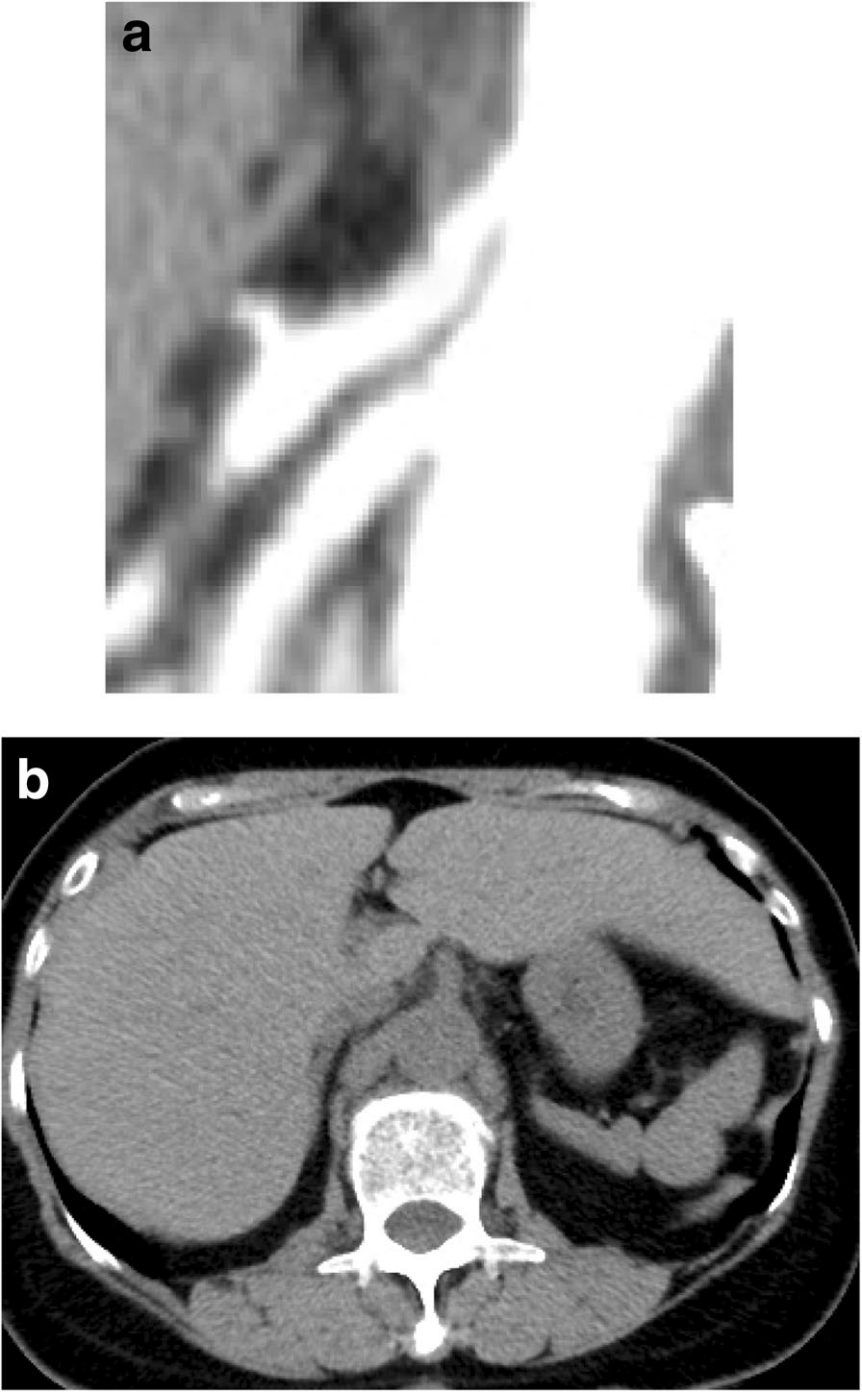
Preoperative multidetector CT showing neither median arcuate ligament syndrome nor arteriosclerosis around the celiac artery. **a** Contrast-enhanced CT. **b** Plain CT

A 69-year-old female presented to our hospital with a one-month history of epigastric discomfort. Physical examination was unremarkable, and laboratory examination was normal other than slightly elevated amylase levels. Abdominal contrast-enhanced computed tomography (CT) revealed a low-density mass with a diameter of 30 mm at the pancreatic head, with segmental superior mesenteric vein (SMV) attachment. There was neither stenosis nor arteriosclerosis around the celiac axis at that time (Fig. 1). Endoscopic ultrasonography was performed, and a fine-needle biopsy sample showed adenocarcinoma of the pancreas.

The patient then underwent PD with superior mesenteric vein resection and reconstruction, and a lymphadenectomy including right half dissection of the lymph nodes without the nerve plexus around the celiac trunk. Intraoperative exploration ruled out latent peritoneal or liver metastasis and showed normal anatomy of the celiac trunk, mesenteric vessels, and related branches. A clamp test of the GDA showed normal hepatic artery pulsation. The pancreatic body needed to be mobilized more than usual for the pancreaticojejunostomy because it was a hard pancreas. The duration of surgery was 549 min, and the blood loss was 863 mL; blood transfusion was not performed.

**Fig. 2:**
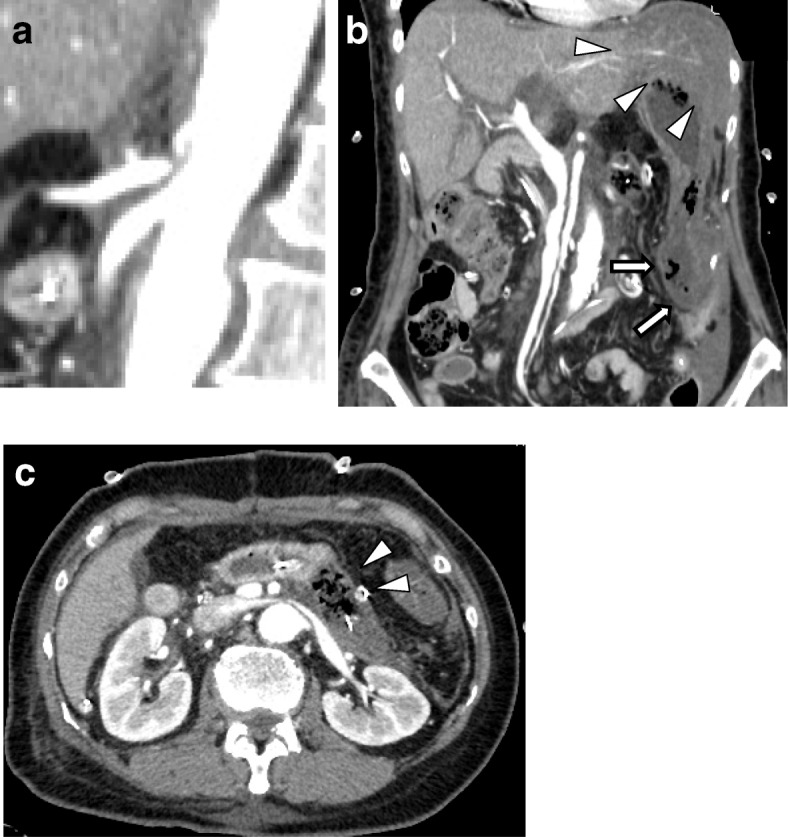
Contrast-enhanced multidetector CT at POD 3 showing an acute extrinsic stenosis caused by median arcuate ligament compression (**a**), ischemia in the liver (arrow head) and the gastrojejunostomy site (arrow) (**b**), and necrosis of the pancreatic body (**c**)

**Fig. 3:**
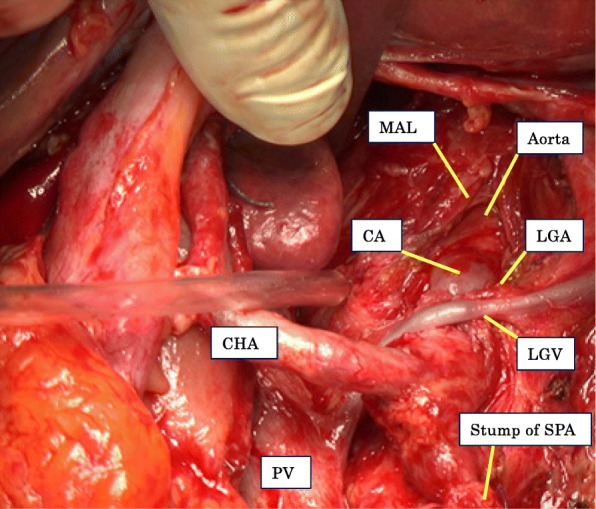
Intraoperative demonstration of median arcuate ligament division

On POD 3, her liver function tests were still abnormal and bloody fluids were found in the drain. Abdominal CT showed a characteristic hook-pattern on the anterior proximal celiac axis from compression of the MAL, which had not been detected in the preoperative CT (Fig. 2a). In addition, ischemic changes in the remnant pancreas, hepatic lateral segment, and gastrojejunostomy site were also observed, although all celiac branches—left gastric artery; splenic artery; common hepatic artery; and right and left hepatic artery—were visible (Fig. 2b, c). We tried interventional radiology first, but the procedure failed due to the tight compression against the outside of the celiac axis. The patient underwent an urgent re-laparotomy. There were almost no pulsations in those celiac branches, showing a markedly decreased blood flow at the celiac axis and stenosis at the root of the celiac artery. No surgery-related damage was detected in any of those branches. Necrosis of the pancreaticojejunostomy site and pancreatic body was also detected. This was considered to be related to the mobilization of the pancreatic body, making this part more susceptible to ischemic damage. The MAL was released, with subsequent dramatic resumption of pulsation in the celiac artery and the common hepatic artery (Fig. 3). Resection of the remnant pancreas and splenectomy was also performed.

**Fig. 4:**
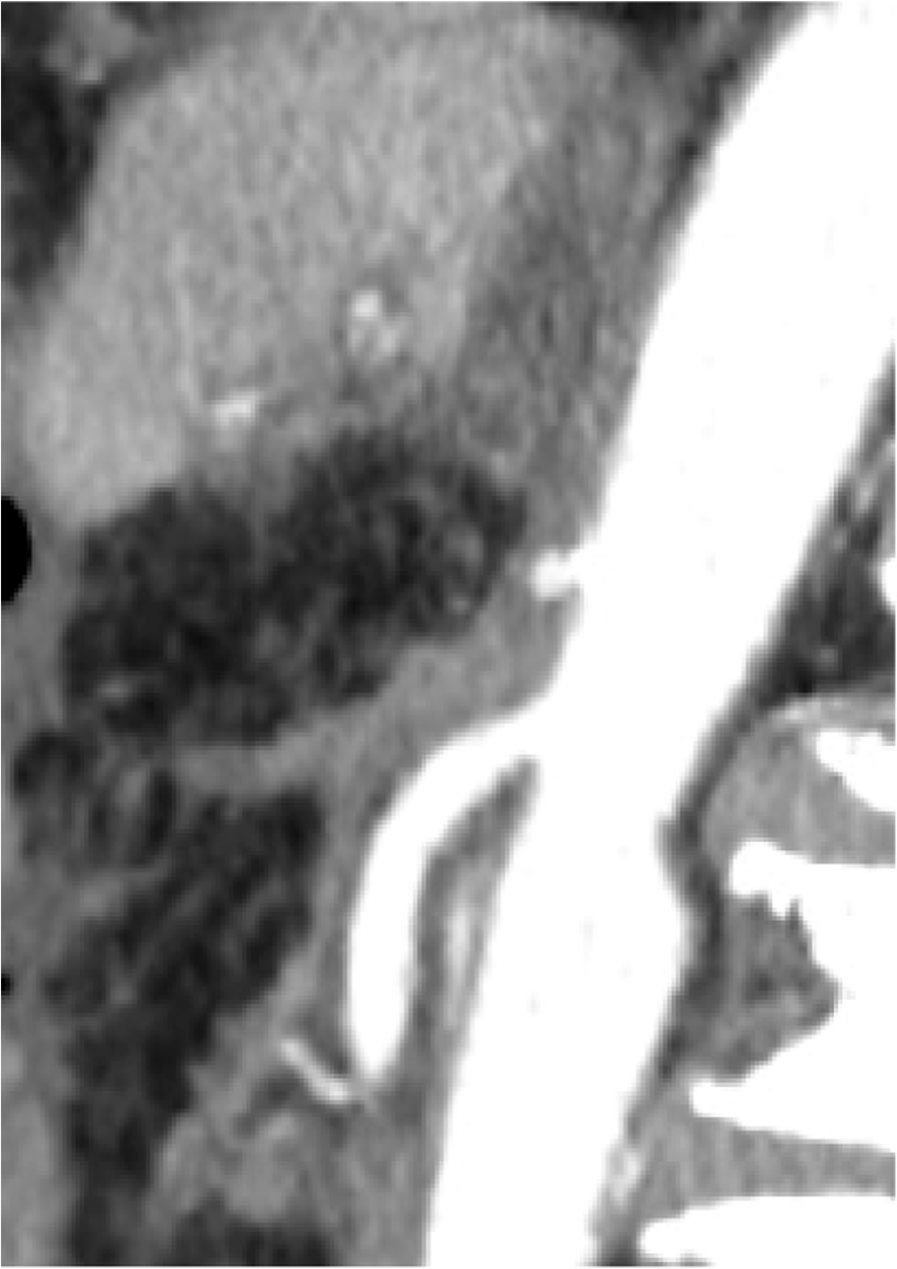
Contrast-enhanced multidetector CT at POD 20 showing occlusion of the celiac artery

**Fig. 5:**
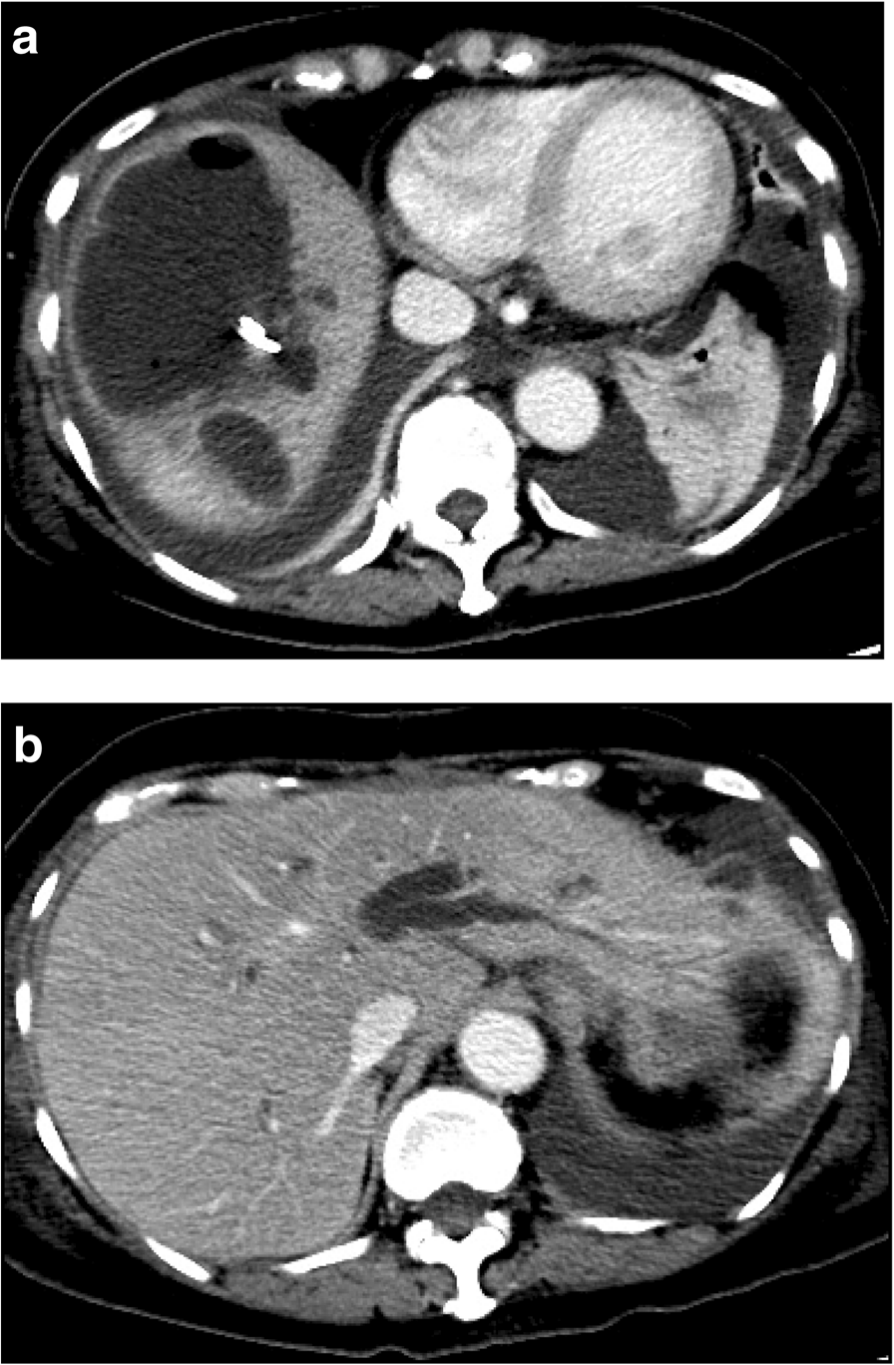
Contrast-enhanced multidetector CT at POD 65 showing severe hepatic cytolysis (**a**) and choledochojejunostomy stenosis (**b**)

Abdominal CT on POD 20 showed re-occlusion of the celiac artery at its root, which was almost same as the first stenotic site (Fig. 4). However, although the right and left hepatic arteries and the left gastric artery were visible, taking from collaterals, the patient experienced rupture of the gastrojejunostomy site, severe hepatic cytolysis and choledochojejunostomy stricture thereafter (Fig. 5a, b). She underwent percutaneous drainage for each. She was discharged to her home at POD 216 with a percutaneous transhepatic cholangial drainage tube.

## Discussion

Celiac artery patency is vital to successful outcome after PD; impairment of celiac flow results in potentially catastrophic hepatic, biliary, gastric, splenic, and pancreatic ischemia [9]. The preoperative diagnosis of MALS is essential so that this arcade may be preserved [5]. Three-dimensional CT angiography shows a characteristic hook pattern on the anterior proximal celiac axis when it is compressed by the MAL [10]. Gaujoux et al. reported that multidetector CT, especially its lateral views, can detect significant arterial stenosis with 96% sensitivity and determine the etiology of celiac axis stenosis with 92% accuracy [11]. If MALS is diagnosed before PD, various methods are available for revascularization before or during the procedure; these include open or laparoscopic MAL division, vascular bypass procedure, or endovascular stenting [7, 8, 12, 13]. Sharafuddin et al. reported 25 cases treated with stent-assisted angioplasty for stenosis of the celiac artery or the superior mesenteric artery with a 96% success rate [7].

Intraoperative assessment of the flow through the hepatic artery should be performed in pancreatic resection, even if preoperative CT does not demonstrate MALS [13]. However, this may not be reliable. Hemodynamically significant stenosis during the GDA clamping test has been reported to be present in about 40% of cases [11]. If MALS is diagnosed during PD, the MAL must be divided at the beginning of the procedure, before GDA ligation or pancreatic division [11, 13]. This safe and fast procedure permits trunk decompression and resolution of ischemic disorders in up to 87% (20 out of 23 cases) of patients [11]. They reported only 1 case with celiac axis occlusion even after MAL division, in which they considered that an ineffective MAL division resulted in celiac axis fibromuscular dysplasia. In this current case, we confirmed a dramatic resumption of the blood flow through the celiac axis and the hepatic artery after MAL division. Therefore, we considered that an effective MAL division could be performed. However, even after such an effective procedure, the celiac axis exhibited re-occlusion, causing many complications. We considered that the radiological procedure before MAL division might have caused the re-occlusion of the celiac axis in this patient. In this radiological procedure, even cannulation of the catheter did not go smoothly because of the tight stenosis of the celiac artery. This procedure might have damaged the endothelial cells of the celiac artery, causing the subsequent re-occlusion. The CT at POD 20 showed that re-occlusion of the celiac artery had occurred near its root, where the stenosis had been. This fact may support our suggestion. We cannot deny the possibility that the MAL might not have been completely released and might have caused the re-occlusion of the celiac artery. In fact, the aortic wall was visible after division of the MAL (Fig. 3). And, we were sure that the celiac stenosis had been completely released at that time because there was significant resumption of the blood flow after the procedure.

MALS can develop acutely after PD even if it has not been diagnosed pre- or peri-operatively. This is the third case which showed no evidence of hepatic artery flow impairment before PD or during GDA clamping or pancreatic transection [4, 13]. There is no known explanation for this acute onset of MALS after PD in a patient with normal celiac anatomy. However, we considered, like two other authors, that this phenomenon might be induced by pre-existing non-significant celiac axis stenosis, with a very tight median arcuate ligament that is exacerbated by lymphadenectomy of the celiac region.
